# Editorial: Targeting oxidative stress in cancer: what is new in the prevention, diagnostic, treatment and prognostic strategies?

**DOI:** 10.3389/fphar.2023.1229785

**Published:** 2023-06-19

**Authors:** Qianjin Liao, ZhiBin Wang, Silvia Maria Suter Correia Cadena

**Affiliations:** ^1^ Hunan Key Laboratory of Cancer Metabolism, Hunan Cancer Hospital, The Affiliated Cancer Hospital of Xiangya School of Medicine, Central South University, Changsha, Hunan, China; ^2^ Public Service Platform of Tumor Organoids Technology, Hunan Gynecological Tumor Clinical Research Center, Changsha, Hunan, China; ^3^ Department of Biochemistry and Molecular Biology, Federal University of Paraná, Curitiba, Paraná, Brazil

**Keywords:** cancer, oxidative stress, antioxidant biomarkers, oxidant biomarkers, ROS

Cancer is one of the leading causes of death worldwide, accounting for about 10 million deaths in 2020. Its incidence increases yearly, compromising individuals, families, and communities physical and emotional health. It is estimated that in 2040 there will be around 30 million cases, an increase of 57% compared to 2020 (WHO-IARC, 2023). Oxidative stress plays an essential role in cancer development and progression since high levels of reactive oxygen species (ROS) can trigger damage to biomolecules, promoting carcinogenesis (Cheung and Vousden, 2022). On the other hand, this metabolic imbalance may result in cell death by different mechanisms, becoming the induction of oxidative stress a potential strategy in anticancer therapy (Luo et al., 2022; Coradduzza et al., 2023).

Considering the challenges in establishing effective cancer therapies and the important role that oxidative stress plays in its development and progression, this Research Topic reunited works that significantly contribute to advance in this research field. Alshehri et al. show the antioxidant, anticancer, and anti-inflammatory activities of ethanolic extracts of *Adenium obesum*, popularly known as “desert rose,” used for the treatment of several diseases. The authors characterized 26 phytochemical compounds from the leaf extract of *A. obesum* related to antioxidant activity evaluated for different methods. Noteworthy, the extract toxicity on tumoral cells was confirmed by the decrease in viability, fragmentation and nuclear condensation of A549 lung cancer cells. In addition, the potential anti-inflammatory activity of the extract was evidenced by the reduction in the levels of main inflammatory mediators in murine alveolar macrophages after treatment with the extract. These significant results contribute to further studies aiming to formulate herbal-based medicine.

In a study elegantly designed, Zhang et al. demonstrated the anti-glioma activity of new juglone derivatives, an antitumor pigment used for years in herbal medicine. The authors performed *in vitro* and *in vivo* experiments using U87 and 251 cell lines and human glioblastoma cells in mice orthotopic model, respectively. The derivatives were more stable than natural juglone and less susceptible to oxidation, preserving the antitumoral activity. Derivatives with allyl or butyl substitution were most effective, inhibiting the proliferation and inducing apoptosis of glioma cells mediated by ROS.

This Research Topic also includes the important work of Tang et al. Under another approach, the authors demonstrated that the hepatoxicity of Sunitinib, a multi-targeted tyrosine kinase inhibitor with remarkable anticancer activity, may be protected by glycyrrhetinic acid. Sunitinib reduced the viability of nontumoral hepatocytes mediated by the activation of mitogen-activated proteins kinases (MAPKs) due to the exacerbation of ROS production. The apoptosis and autophagy induced by Sunitib were relieved by the treatment with glycyrrhetinic acid. According to the authors, glycyrrhetinic acid could be a preventive therapy to reduce liver injury caused by Sunitinib.

In a study characterized by a robust experimental design, Rahimifard et al. demonstrated the synergistic effects of cisplatin-resveratrol combination in inhibiting metastasis and promoting apoptosis and cellular senescence through modulation of P38/P53 and P16/P21 pathways. The co-administration of cisplatin and resveratrol resulted in an increase in ß-galactosidase activity, ROS levels as well as upregulation of p53, p38, p16, p21, and MMP-2 gene expression, leading to arrest of cell cycle at G0/G1 phase. Furthermore, the treatment suppressed telomerase activity, pro-inflammatory gene expression, and cell invasion. These findings highlight the potential clinical utility of cisplatin-resveratrol combination in the management of cancer.

This area of research also encompasses the important contributions made by Liu et al. Platinum-based chemotherapy induces cancer cell death by elevating oxidative stress levels to a cytotoxic extent. Liu et al. have identified a noteworthy association between the CAT rs769217 polymorphism and platinum-based chemotherapy-related progression-free survival in patients with lung cancer, indicating its potential as a prognostic biomarker for such patients. The CAT gene encodes catalase, a key antioxidant enzyme that modulates reactive oxygen species and is critical in the body’s defense against oxidative stress.

A pioneering review by Li et al. has uncovered the importance of ROS in regulating multiple signaling pathways. Cancer cells can resist therapy by increasing their antioxidant defense system to cope with high levels of ROS. The authors summarized the molecular mechanisms behind this resistance, including drug efflux, DNA repair, stemness maintenance and tumor microenvironment alteration.

Zhuo et al.’s significant contribution to the field must also be acknowledged. They demonstrated that the oncogene eIF3a plays a crucial role in cancer development and responses to various therapies, especially those known to promote oxidative stress. Using a proteomics approach, they systematically elucidated its relationship with oxidative stress and found that it is involved in lipid peroxidation, which affects the response of cancer cells to cytotoxic antitumor drugs. These findings suggest that eIF3a may serve as a bridge between oxidative stress and cancer, providing insights into cancer development and therapy from cellular processes, molecular signaling pathways, metabolism, and immune responses.
